# Genetic Polymorphisms in Homocysteine Metabolism and Response to Folate Intake: A Comprehensive Strategy to Elucidate Useful Genetic Information

**DOI:** 10.2188/jea.JE20100042

**Published:** 2010-07-05

**Authors:** Koichi Miyaki

**Affiliations:** Division of Clinical Epidemiology, Department of Clinical Research and Informatics, National Center for Global Health and Medicine, Tokyo, Japan

**Keywords:** homocysteine, metabolism, folate, single nucleotide polymorphism (SNP), genome wide association study (GWAS)

## Abstract

Homocysteine is a risk factor for atherosclerosis, and the level of homocysteine in plasma is known to be strongly influenced by genetic factors—not only rare variants, but also common polymorphisms. This report describes a comprehensive postgenomic strategy for elucidating useful genetic information about homocysteine metabolism. The standard method for gathering such information is the candidate gene approach, which is an effective method based on known biological information. After collecting evidence from independent research projects, a critical epidemiological review permits a determination as to whether a putative association is true or not. A genome-wide association study (GWAS), which requires no biological information, can identify new candidates and confirm associations suggested by the candidate gene approach. The importance of methylenetetrahydrofolate reductase (MTHFR) C677T polymorphism, which was shown in a randomized controlled trial conducted by the present author, and in other studies, was independently confirmed by a large-scale GWAS. GWASs have also identified new candidate genes, but these must be confirmed by independent studies. In homocysteine metabolism, the classical candidate gene approach was sufficiently robust to detect the true association. However, candidate markers newly discovered by GWAS need to be confirmed by well-designed epidemiological studies to determine their significance. International statements, such as CONSORT and STREGA, provide useful principles for conducting such research.

## INTRODUCTION

Several novel risk factors for atherosclerosis have been proposed by Ridker et al^1^; one of these is homocysteine, an amino acid found in plasma. McCully firstly reported the vascular pathology of severe inherited homocysteinemia, in 1969.^2^ Since then, hyperhomocysteinemia has been extensively studied, and elevated plasma total homocysteine (tHcy) level has been identified as a risk factor for atherosclerotic vascular diseases in many persuasive systematic reviews.^3^^–^^5^

An important determinant of plasma homocysteine concentration is dietary intake of folate, which enhances the importance of homocysteine as a biomarker because the level of homocysteine can be lowered by eating more green vegetables and by folic acid supplementation.^6^^,^^7^

Investigations at the genetic level have also progressed, and have identified not only rare mutations that cause severe inherited homocysteinemia, but also common polymorphisms that result in moderate elevation of homocysteine level.

This short report presents a comprehensive strategy to elucidate the useful genetic information in the postgenomic era, using the example of homocysteine metabolism.

### MTHFR C677T polymorphism

**Figure 1.**
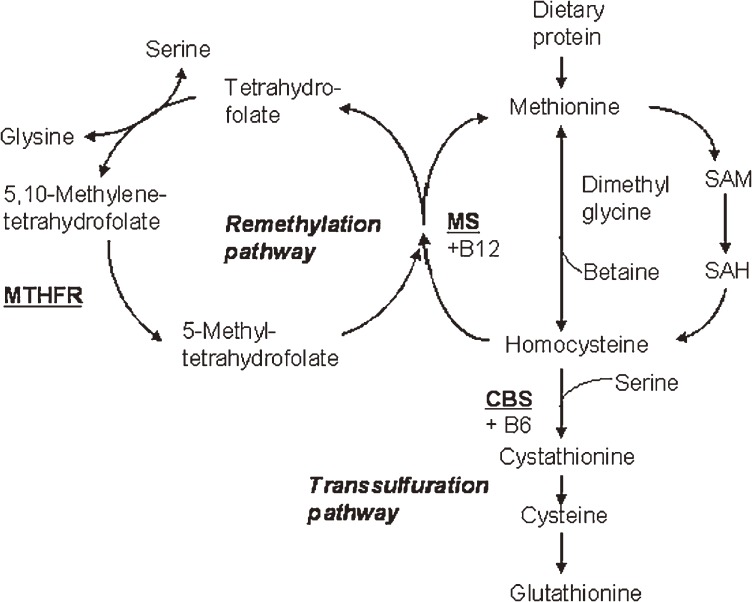
The metabolic pathway of homocysteine. (Adapted from Fowler B Disorders of homocysteine metabolism. J Inherit Metab Dis 1997)

A candidate gene approach, based on known biological information, is an established and effective method of genetic research. It is natural to focus on the gene involved in the biological mechanisms of the target phenotype. For example, in the metabolic pathway of homocysteine, there are 3 key enzymes: methylenetetrahydrofolate reductase (MTHFR), methionine synthase (MS), and cystathionine beta-synthase (CBS) (Figure 1).

MTHFR is a key enzyme in the metabolic pathway of homocysteine. Single nucleotide polymorphisms (SNPs) have been identified in the genes of related enzymes. The present author investigated these candidate SNPs and focused on a common C to T substitution at position 677 of the MTHFR gene (MTHFR C677T polymorphism), which results in an alanine to valine amino acid substitution in the protein and leads to a 30% decrease in enzyme activity in heterozygotes and a 60% decrease in homozygotes.^8^ This TT genotype was known to cause mild hyperhomocysteinemia more frequently than the major allele homozygote, and its allele frequency approaches 30% in many ethnic groups.^9^

### Randomized controlled trial examining each MTHFR C677T genotype

In a previous study,^10^ the present author investigated the MTHFR C677T polymorphism, which is common and functional^8^^,^^9^; 210 healthy males were enrolled in the study. After excluding those who were taking folic acid or drugs known to effect folic acid metabolism, 203 healthy males remained. The mean age and body mass index (BMI) were 45.8 ± 11.5 years and 23.7 ± 3.66 kg/m^2^ (mean ± SD), respectively, which are typical values for healthy Japanese male workers. Genotyping for the MTHFR C677T polymorphism was performed by using a polymerase chain reaction (PCR) technique and restriction fragment length polymorphism (RFLP) analysis. The intervention in this randomized controlled trial (RCT) consisted of oral folic acid 1 mg/day, or an identical-looking placebo, for 3 months, because reduction in total homocysteine is known to be maximal at a folic acid dosage of 1 mg/day (one study reported that a minimum of 0.8 mg/day was necessary to achieve maximum reduction in tHcy level).^6^

The genotyping results were in Hardy-Weinberg equilibrium and were consistent with results previously reported for another Japanese population.^11^^,^^12^ Among the participants receiving folic acid, there was a significant difference in the decrease in plasma tHcy between MTHFR C677T genotypes.^10^ The TT homozygote group showed the largest decrease in plasma tHcy at both 1 month and 3 months after starting the intervention. Based on consistent results observed twice during the course of the intervention, the effect size of the tHcy reduction in TT homozygotes was estimated to be 2.4 times that of the wild type. There were significant linear trends between the allele number and the decrease in plasma tHcy at 1 month and 3 months (*P* < 0.01 for both). The results of these trend tests remained statistically significant after adjustment for age, BMI, smoking status, and alcohol consumption at 1 month and 3 months (*P* < 0.01 for both). Thus, in an RCT prepared in accordance with the Consolidated Standards of Reporting Trials (CONSORT) statement,^13^ MTHFR C677T genotype was shown to influence the plasma level of homocysteine and the response to folic acid intake.

### Other SNPs

Our previous study showed that the MTHFR C677T minor allele homozygote was associated with a 2.4-fold beneficial decrease in plasma homocysteine level, as compared with the other genotypes. Next, we performed additional genotyping over the whole sequences of this gene. Of 52 SNPs on the MTHFR gene, 4 SNP loci encompassing more than 80% of the relevant information were selected, and haplotypes were estimated. The haplotypes were classified into 3 groups (Hap0, Hap1, and Hap2) on the basis of the number of times the most frequent haplotype was present. The decrease was greater in Hap2 (6.61 µmol/L) than in the other haplotypes (Hap0, 2.67; Hap1, 2.60; trend test, *P* < 0.01).^14^ The haplotype information obtained was not more informative than that obtained by grouping according to the single SNP C677T, which strongly influences enzyme activity. This indicates that, from the perspective of informatics, increasing the number of typed SNPs does not necessarily provide more information.

### Genome-wide association studies

Recently, a reliable genome-wide association study (GWAS) of homocysteine was published,^15^ and the influence of MTHFR C677T polymorphism was clearly confirmed using an independent method. Although the effect of MTHFR C677T polymorphism had been shown in a well designed study, it was nevertheless desirable to confirm the findings, using a methodologically independent approach.

From a methodological standpoint, GWASs are ideal, because they employ a purely statistical approach that requires no biological information. Rapid advances in genotyping technology have made it possible to use a large number of genetic markers in a straightforward way. GWASs have become increasingly used in the United Kingdom and the United States. This method involves identifying genetic variations associated with a particular disease by screening markers across the complete genomes of many individuals. Confirmation of the findings, using a different method, enhances the accuracy of the results.

**Figure 2.**
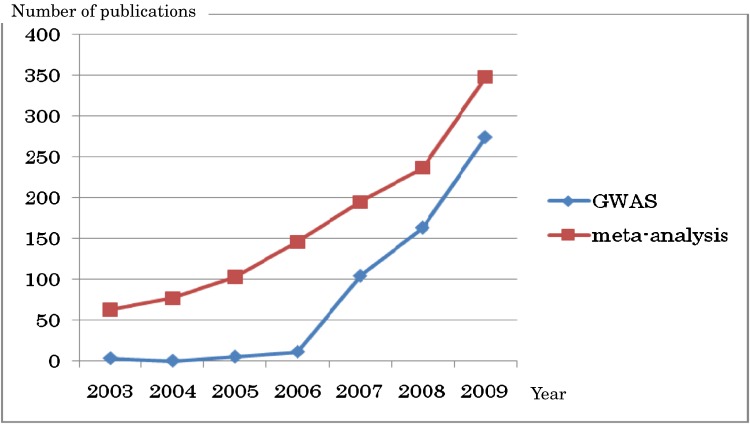
Trends in the numbers of articles on genomic meta-analysis and GWAS in 2003–2009 (as of Feb 23, 2010). GWAS, Genome-wide association study.

**Figure 3.**
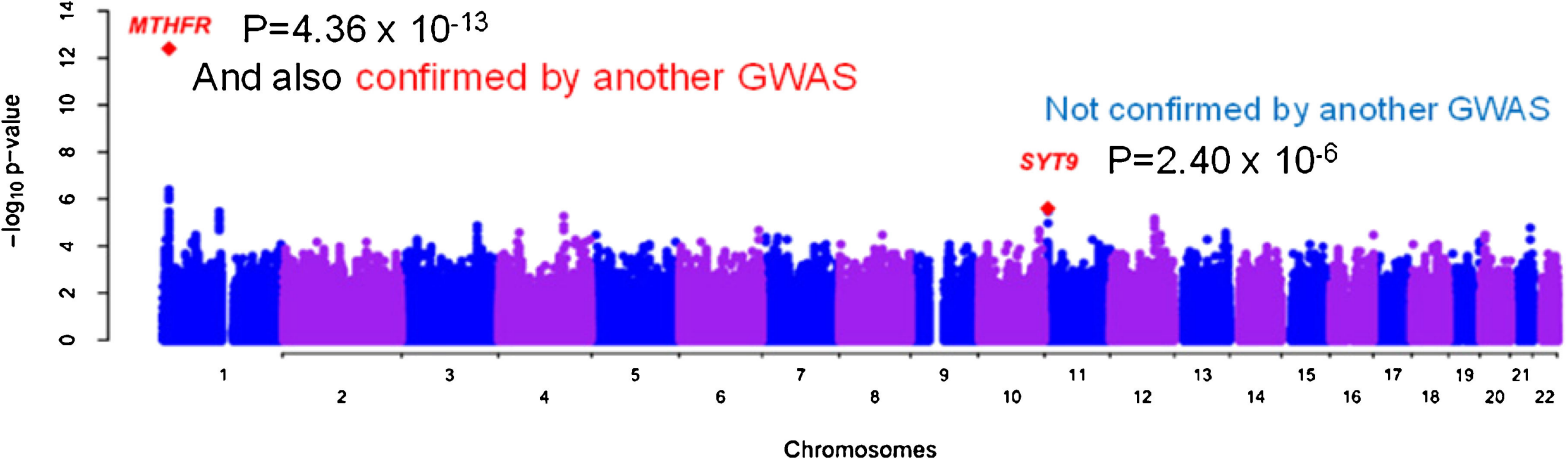
Genome-wide scan of plasma homocysteine graphed by chromosome position and −log_10_ *P*-value. (Adapted from Tanaka T Genome-wide Association Study of Vitamin B6, Vitamin B12, Folate, and Homocysteine Blood Concentrations. ***Am J Hum Genet*** 2009)

Since 2003, during which the first 3 GWASs were reported, the number of articles on genomic meta-analysis and the GWAS has been increasing (Figure 2). There are 274 GWAS reports in the HuGE Navigator database for 2009. Among these was the first reliable report of a genome-wide scan of homocysteine.^15^ Figure 3 shows the genome-wide association of plasma homocysteine graphed by chromosome position and log-transformed *P* value. The most significant variant was in the MTHFR gene, on chromosome 1. This variant reached genome-wide significance with a Bonferroni-corrected threshold. This is the C677T polymorphism itself, the significance of which was shown in our RCT using the candidate gene approach.

## DISCUSSION

The importance of the MTHFR C677T SNP was confirmed in a large-scale, methodologically independent, GWAS. After collecting and reviewing all relevant research, a critical epidemiological review of the evidence can help to assess whether a putative association is true or not. The GWAS approach, which requires no biological information, can identify new candidates and confirm associations suggested by the candidate gene approach.

**Figure 4.**
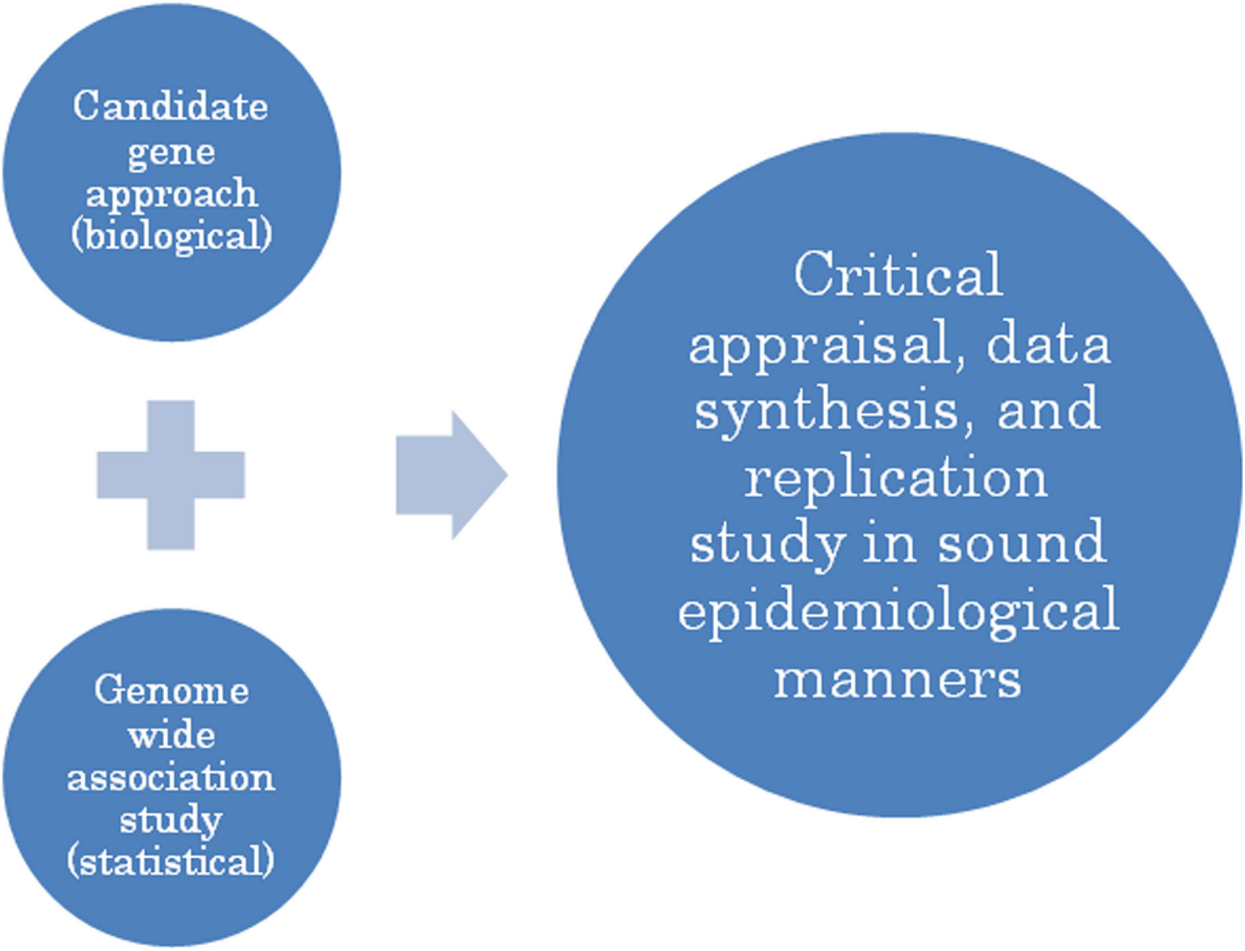
A proposed comprehensive strategy for elucidating useful genetic information in the postgenomic era.

Figure 4 shows a proposed strategy for collecting useful genetic information in the postgenomic era. With respect to homocysteine metabolism, as indicated in this article, a classical candidate gene approach is sufficiently robust to detect a true association between genotype and phenotype. However, candidate markers newly discovered by GWASs require confirmation by well designed epidemiological studies. The present author uses such a strategy, in part because the considerable resources required by a whole-genome scan are simply not necessary. The results of GWAS can be used as a kind of high-priced screening to design a focused epidemiological study.

Recently, the *Strengthening the Reporting of Genetic Association Studies* (STREGA) initiative was introduced to extend the *Strengthening the Reporting of Observational Studies in Epidemiology* (STROBE) Statement, and provides additions to 12 of the 22 items on the STROBE checklist.^16^^–^^22^ The publication of these guidelines in major journals is important because I believe sound epidemiological design should be emphasized in genetic studies. As Thelle has pointed out, the STREGA checklist does not itself provide new ideas, but instead emphasizes the need for systematic and transparent reports using guidelines that, although well known to epidemiologists, may not be widely known among molecular biologists and geneticists.^23^ Using such guidelines, the quality of genetic association studies is likely to increase, and better reporting of such studies would facilitate the synthesis of evidence from different reports. This synthesis of knowledge is crucial if evidence-based genomics is to be integrated with the practice of medicine and public health. In this context, I believe that epidemiology is needed by genomics, even if not vice versa.

The US Institute of Medicine released a report regarding the future of public health.^24^ They underscored the need for a broad-based ecological approach to human health and identified 8 specific content areas that are needed to address new challenges (the first two of these areas are informatics and genomics). Although human genome studies are still in the development stage, I would like to contribute to public health by encouraging epidemiological studies that highlight new techniques in genomics and informatics.
